# Dual Lumen Microcatheter in Percutaneous Biliary Drainage for Postoperative Bile Leakage: A Case Report

**DOI:** 10.7759/cureus.49274

**Published:** 2023-11-23

**Authors:** Yasuyuki Onishi, Hironori Shimizu, Yuki Masano, Takashi Ito, Yuji Nakamoto

**Affiliations:** 1 Diagnostic Imaging and Nuclear Medicine, Kyoto University, Kyoto, JPN; 2 Surgery, Kyoto University, Kyoto, JPN

**Keywords:** post-operative complication, living donor liver transplantation, dual lumen microcatheter, percutaneous biliary drainage, bile leak

## Abstract

Percutaneous biliary intervention is widely accepted as an effective and safe treatment for various types of bile duct diseases. We present the case of a 44-year-old woman who developed bile leakage after a living-donor liver transplantation for locally advanced cholangiocarcinoma. A percutaneous drainage tube was placed in the segment 8 bile duct via the blind end of the jejunum. However, the bile leakage was unchanged. Bile leakage from the right posterior hepatic duct was suspected. Using a dual lumen microcatheter, a percutaneous drainage tube was placed in the segment 7 bile duct via the blind end of the jejunum, which reduced the bile leakage. These results suggest that a dual lumen microcatheter is a valuable tool for navigating the biliary tree during difficult percutaneous biliary interventions.

## Introduction

Percutaneous biliary intervention is a useful treatment for various bile duct diseases [1]. During the procedure, 4- or 5-F angiographic catheters and 0.035-inch guidewires are commonly used to navigate the bile ducts [2,3]. However, in difficult cases, angiographic catheters and 0.035-inch guidewires are insufficient. A dual lumen microcatheter (DLM) has two lumens that enable independent manipulation of two guidewires [4]. The DLM has been used for percutaneous coronary interventions for bifurcation lesions and chronic total occlusions [4-6]. To our knowledge, no previous study has reported its use during percutaneous biliary interventions. This report presents a patient with postoperative bile leakage in whom percutaneous biliary drainage was successfully performed using a DLM.

## Case presentation

A 44-year-old woman underwent a living-donor liver transplantation for locally advanced cholangiocarcinoma. A right lobe liver graft was used, and the right hepatic duct was anastomosed with the jejunum. Nineteen days after the liver transplantation, blood tests revealed elevated levels of liver enzymes (Table 1).

**Table 1:** Blood test results performed on postoperative days 18 and 19. POD, postoperative day; AST, aspartate transaminase; ALT, alanine transaminase; LDH, lactate dehydrogenase; ALP, alkaline phosphatase; γ-GT, γ -glutamyl transpeptidase; CRP, C-reactive protein

Test	Reference range	POD 18	POD 19
White blood cells	3.3–8.6 × 10^3^/uL	10.6 × 10^3^/uL	13.6 × 10^3^/uL
Hemoglobin	11.6–14.8 g/dL	9.0 g/dL	9.4 g/dL
Platelets	158–348 × 10^3^/uL	497 × 10^3^/uL	520 × 10^3^/uL
AST	13–30 U/L	71 U/L	818 U/L
ALT	7–23 U/L	66 U/L	733 U/L
LDH	124–222 U/L	273 U/L	435 U/L
ALP	38–113 U/L	271 U/L	305 U/L
γ-GT	9–32 U/L	496 U/L	521 U/L
Total bilirubin	0.4–1.5 mg/dL	0.8 mg/dL	0.8 mg/dL
CRP	≤0.14 mg/dL	3.94 mg/dL	4.53 mg/dL

**Figure 1:**
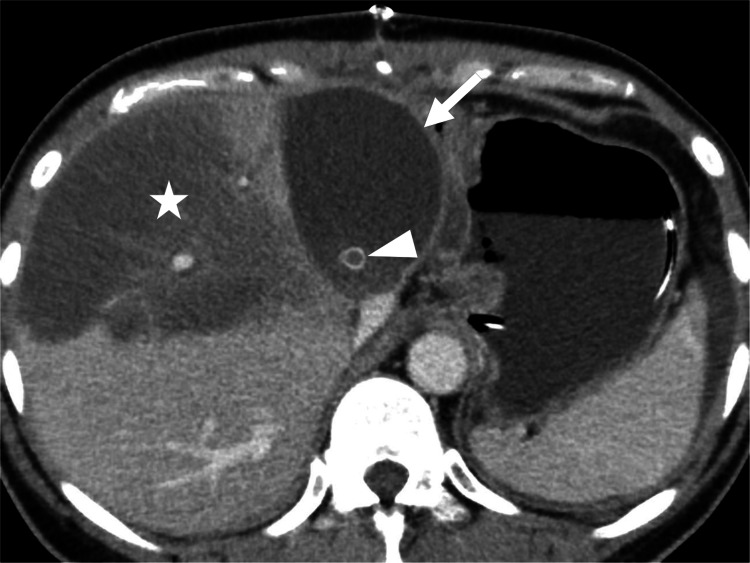
Contrast-enhanced CT of the upper abdomen showing a biloma (arrow) adjacent to the right lobe liver graft; the anterior portion of the liver shows hypodensity (star) due to occlusion of a segmental hepatic vein (arrowhead) that was reconstructed using an artificial graft.

Contrast-enhanced computed tomography (CT) revealed a biloma and obstruction of the right anterior segmental hepatic veins that were reconstructed using artificial grafts (Figure 1).

**Figure 2:**
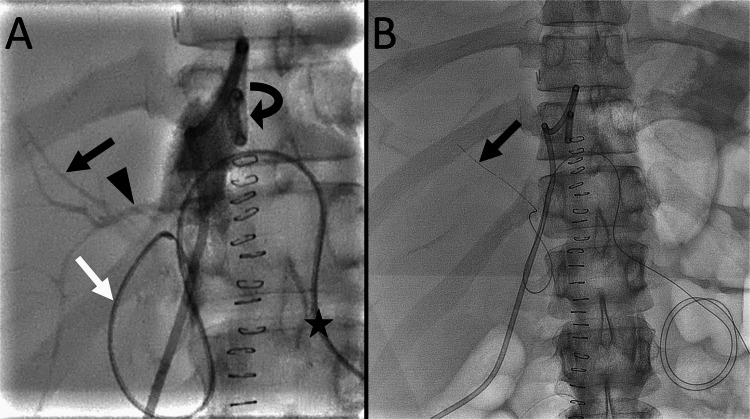
Percutaneous biliary drainage tube placement in the right anterior segmental bile duct (A) A frontal radiograph with a contrast injected through the drainage tube (curved arrow) in the biloma shows the segment 8 bile duct (black arrow) and the right posterior hepatic duct (arrowhead). A seeking catheter (white arrow) is advanced from the blind end of the jejunum. The catheter is inserted through the blind end of the jejunum (star). (B) A frontal radiograph shows a drainage tube (arrow) placed in the segment 8 bile duct.

On CT, the percutaneous bile duct drainage tube placed through the blind end of the jejunum during liver transplantation was dislodged from the bile duct. A percutaneous drainage tube was placed to drain the biloma. The output from the drainage tube was 400 mL/day, and a postoperative bile leakage was suspected. Endoscopic biliary drainage was considered difficult due to postoperative intra-abdominal adhesions. Percutaneous transhepatic biliary drainage was believed to be difficult as the intrahepatic bile ducts were not dilated. Additionally, the transhepatic approach was associated with a risk of right posterior segmental hepatic artery injury, which can be life-threatening. Therefore, a bile duct drainage tube was placed via the blind end of the jejunum by exchanging the dislodged drainage tube. The procedure was performed under local anesthesia and moderate sedation. The dislodged tube was then replaced with a 4-F seeking catheter. Contrast injection from the drainage tube within the biloma revealed the bile ducts (Figure 2A). After careful manipulation of the catheter and a 0.035-inch hydrophilic guidewire, the right anterior hepatic duct was selected. Subsequently, a 4-F, straight-type drainage tube was placed in the segment 8 bile duct (Figure 2B).

**Figure 3:**
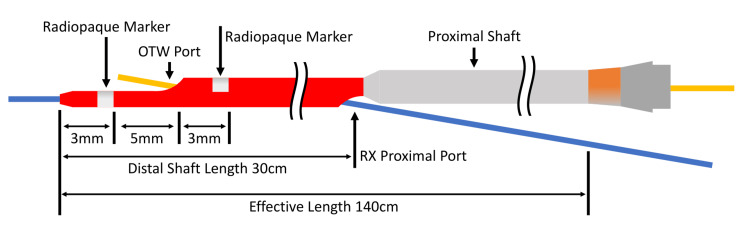
Crusade catheter The catheter has an over-the-wire (OTW) lumen and a rapid-exchange (RX) lumen. There are two radiopaque markers at the distal end of the catheter, and the OTW port is located between the two markers. The distal and proximal shaft diameters are 2.9-F and 3.2F. Image credit: Authors

However, the output from the biloma drainage tube remained unchanged. A bile leakage from the right posterior hepatic duct was suspected, and a drainage tube was placed in the right posterior segmental bile duct nine days after the first percutaneous biliary intervention. After exchanging the drainage tube with a 4-F seeking catheter, the advancement of a 0.035-inch hydrophilic guidewire to the right posterior hepatic duct was attempted, though it was not possible as the catheter was unstable at the hepaticojejunal anastomosis site. Therefore, a Crusade catheter (Crusade Type R; Kaneka Corporation, Minato City, Tokyo, Japan), a popular DLM in Japan (Figure 3), was used.

**Figure 4:**
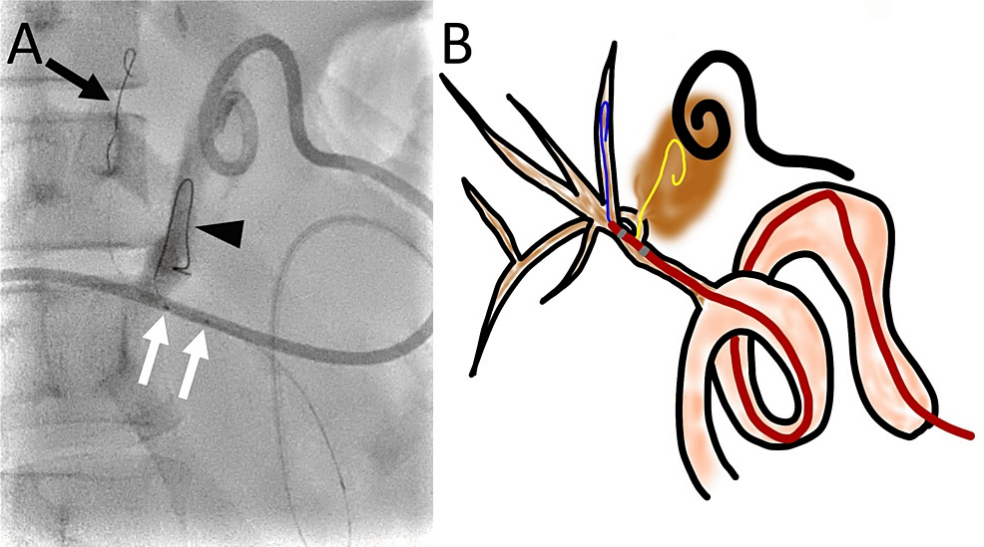
Advancement of a guidewire to the biloma through the defect at the right posterior hepatic duct using a Crusade catheter (A) A right oblique radiograph shows the advancement of one guidewire (arrow) to the segment 8 bile duct and another guidewire (arrowhead) to the biloma. The two markers of the Crusade catheter are observed (white arrows). (B) A schematic drawing shows a guidewire (blue) that was advanced to the segment 8 bile duct, which was used as a safety wire. The Crusade catheter is advanced on the first guidewire along the rapid-exchange lumen. The position of the Crusade catheter is adjusted to advance the second guidewire (yellow) to the right posterior hepatic duct through the over-the-wire lumen. The second guidewire (yellow) is then advanced to the biloma (brown) through the defect at the right posterior hepatic duct. Image credit: Authors Manufacturer details: Crusade catheter; Kaneka Corporation, Minato City, Tokyo, Japan

A 0.014-inch guidewire was advanced to the segment 8 bile duct as a safety wire, and the seeking catheter was removed. A Crusade catheter was advanced to the hepaticojejunal anastomosis along the 0.014-inch guidewire through the rapid-exchange lumen. Another 0.014-inch, 300-cm-long guidewire was advanced through the over-the-wire lumen to select the right posterior hepatic duct. The second guidewire was advanced into the biloma through a fistula at the proximal right posterior hepatic duct (Figures 4A, 4B).

**Figure 5:**
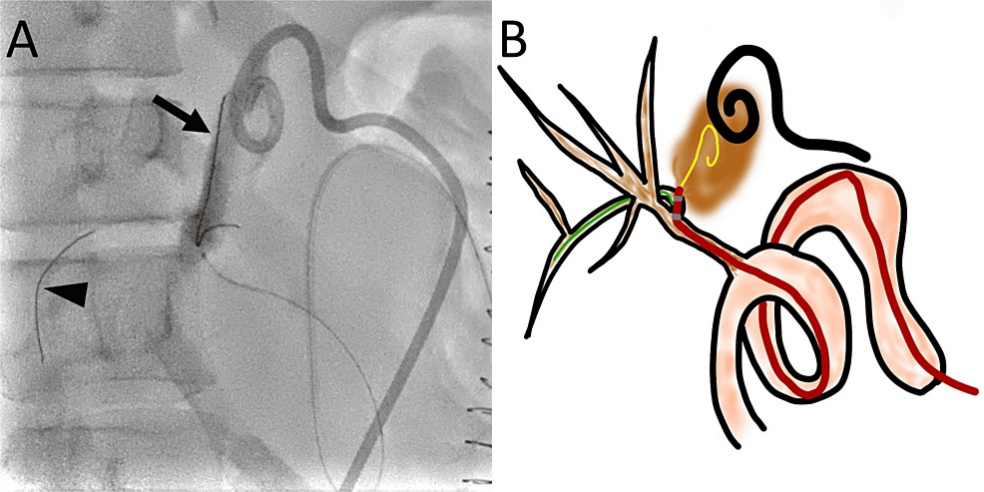
Advancement of a guidewire to the right posterior segmental bile duct using a Crusade catheter (A) A right oblique radiograph shows one guidewire (arrow) that is advanced to the biloma. The other guidewire (arrowhead) is advanced to the segment 6 bile duct. (B) A schematic drawing shows the guidewire (yellow) that was advanced to the biloma and used as a safety wire. The Crusade catheter is advanced using the guidewire along the rapid-exchange lumen. The position of the Crusade catheter is adjusted to advance another guidewire (green) to the right posterior segmental bile duct. Subsequently, the guidewire is advanced to the segment 6 bile duct. Image credit: Authors Manufacturer details: Crusade catheter; Kaneka Corporation, Minato City, Tokyo, Japan

Advancing the guidewire to the right posterior segmental bile duct distal to the fistula was not possible. Therefore, the Crusade catheter was used again to select the right posterior segmental bile duct using the second guidewire as a safety wire. The first guidewire and the Crusade catheter were removed, leaving the second guidewire in place in the biloma. The Crusade catheter was advanced along the second guidewire through the rapid-exchange lumen. Another 0.014-inch, 300-cm-long guidewire was advanced through the over-the-wire lumen, and the segment 6 bile duct was selected (Figures 5A, 5B).

**Figure 6:**
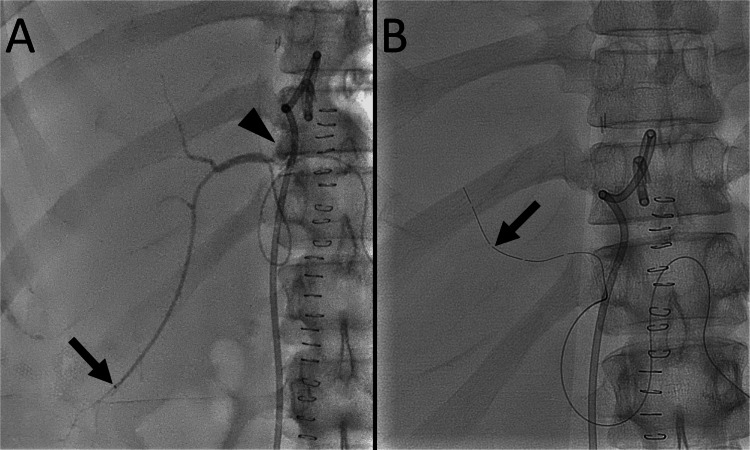
Percutaneous biliary drainage tube placement in the right posterior segmental bile duct (A) A frontal radiograph with contrast injected using a microcatheter (arrow) advanced to the segment 6 bile duct shows leakage of the contrast medium (arrowhead) into the biloma. (B) A frontal radiograph shows a drainage tube (arrow) placed in the segment 7 bile duct.

The Crusade catheter was removed, and a microcatheter was advanced along the guidewire into the segment 6 bile duct. A contrast injection from the microcatheter revealed leakage from the proximal right posterior hepatic duct into the biloma (Figure 6A). Subsequently, a 4-F, straight-type drainage tube was placed in the segment 7 bile duct (Figure 6B).

After the procedure, the output from the biloma drainage tube decreased. Four days after the final procedure, the output ceased.

## Discussion

In this patient, the tip of the seeking catheter was pulled to the hepaticojejunal anastomosis to select the right posterior hepatic duct. As the catheter passed through the jejunum, adjusting the catheter tip position at the anastomotic site was difficult due to the risk of catheter dislodgement from the anastomotic site. When using a Crusade catheter, one guidewire is placed as a safety wire, and the Crusade catheter can be pulled and pushed along the safety wire without the risk of dislodgement. Similarly, it was difficult to adjust the catheter position in the proximal right posterior hepatic duct due to the risk of catheter dislodgement from the right posterior hepatic duct, and the Crusade catheter was used to select the right posterior segmental bile duct. Therefore, the stability of the Crusade catheter along the safety wire played a key role in allowing the wire to advance to the target bile duct.

Similar to the patient in this report, a previous report described a patient in whom a DLM was successfully used to select the right inferior phrenic artery that branched from the left gastric artery for transarterial chemoembolization for hepatocellular carcinoma [7]. In that report, the stability of the DLM along the safety wire that was advanced to the left gastric artery enabled the selection of the right inferior phrenic artery.

However, the use of a Crusade catheter has some disadvantages. First, its proximal shaft diameter is 3.2-F, and it cannot be advanced through a diagnostic catheter. Second, Crusade catheters are much more expensive than angiographic catheters. In addition, two expensive 0.014-inch guidewires are required. Therefore, the use of the Crusade catheter requires careful case selection.

## Conclusions

In most percutaneous biliary intervention, 4- or 5-F angiographic catheters and 0.035-inch guidewires are sufficient for the procedure. However, when wire advancement to the target bile duct is difficult due to the instability of the catheter, DLMs are useful tools for successful wire advancement.
